# Associations of psychological capital, demographic and occupational factors with cigarette smoking among Chinese underground coal miners

**DOI:** 10.1186/s12889-015-1349-6

**Published:** 2015-01-21

**Authors:** Li Liu, Xin Xu, Hui Wu, Yilong Yang, Lie Wang

**Affiliations:** Department of Social Medicine, School of Public Health, China Medical University, 92 North 2nd Road, Heping District, Shenyang, Liaoning 110001 PR China; Department of Clinical Epidemiology, Library, Shengjing Hospital of China Medical University, No.36 Sanhao Street, Shenyang, Liaoning 110004 PR China

**Keywords:** Smoking, Psychological capital, Effort-reward imbalance, Perceived physical environment, Underground coal miners

## Abstract

**Background:**

As a specific male occupational group, underground coal miners have been commonly found to have a high prevalence of cigarette smoking. It is of urgent need to explore some factors that could be intervened to reduce smoking from personal or internal perspective. The purpose of the present study was to examine the associations of psychological capital (PsyCap), demographic and occupational factors with smoking among Chinese underground coal miners.

**Methods:**

A cross-sectional survey was conducted in a coal-mining population in northeast China. Twenty-five hundreds of male underground miners were sampled from six coal mines. Self-administered questionnaires involving current smoking status, specific scales to measure the levels of PsyCap, effort-reward imbalance (ERI) and perceived physical environment (PPE), and some demographic and occupational factors were completed anonymously after a day shift. Complete responses were obtained from 1,956 participants (response rate: 78.2%). Multivariate logistic regression analysis was used to estimate the factors in relation to current smoking.

**Results:**

The overall smoking prevalence was 52.4%. After controlling for demographic and occupational variables, PsyCap was not associated with smoking. Compared with the miners in the lowest tertile of resilience, the odds ratios (ORs) of smoking for the miners in the intermediate tertile and highest tertile were 1.30 (95% confidence intervals (CI): 0.99–1.70) and 1.58 (95% CI: 1.13–2.20), respectively. Compared with the miners in the lowest tertile of optimism, the ORs of smoking for the miners in the intermediate tertile and highest tertile were 0.79 (95% CI: 0.61–1.03) and 0.69 (95% CI: 0.51–0.92), respectively. Low education and high PPE were the risk factors of smoking, whereas ERI had no association with smoking.

**Conclusions:**

More than half of the underground coal miners were current smokers, which indicated that cigarette smoking might be a common health risk behavior in this occupational population. High resilience and PPE, together with low education were the risk factors of smoking, whereas high optimism was a protective factor. Consequently, PsyCap had mixed effects on cigarette smoking. Investment in resilience and optimism should be given more attention for the purposes of the prevention and reduction of smoking among occupational populations.

**Electronic supplementary material:**

The online version of this article (doi:10.1186/s12889-015-1349-6) contains supplementary material, which is available to authorized users.

## Background

As a preventable cause of disability, death and economic loss, cigarette smoking is one of the most important public health challenges worldwide. In particular, the harm originated from tobacco use will continue to expand in some developing countries, due to population growth and inadequate tobacco control measures [1]. China is the largest producer of tobacco and has the most tobacco consumers in the world. Currently, there are more than 300 million current smokers in China, and tobacco use has been responsible for about one million deaths annually [2]. Cigarette smoking has been commonly found to be more prevalent among men (52.9%) than women (2.4%) in China [3]. Furthermore, in males, the highest smoking prevalence has been found among manual workers across occupational groups, such as agricultural workers and machine operators [3,4]. As a male occupational group, underground coal miners have been commonly found to have a high prevalence of smoking behavior, and those who worked at Zonguldak, Karadon and Gelik coal mines in Turkey and worked at two coal mines of Xuzhou Coal Mining Group in China were reported to have 66.3% and 56.3% smoking prevalence, respectively [5,6]. There are strict rules and regulations concerning the safety of workplace in the process of mining production, and the smoking behavior of underground coal miners is absolutely prohibited, but it does not really reduce the prevalence of cigarette smoking in this occupational population. It is possible that smoking behavior has not been effectively controlled after work. In addition to the harm directly linking to cigarette smoking itself, when smoking is combined with other risk factors in underground working environment (such as dust and radon), the risk of various respiratory diseases among underground coal miners could be increased significantly [6,7]. Therefore, it is important for those interested in tobacco control to explore the factors related to cigarette smoking in this occupational population.

Cigarette smoking is a rather complex behavior, both occupationally psychological and social factors play important roles in its pathogenesis among some occupational populations [8,9]. In the workplace, job stress and psychological distress (e.g., anxiety and depression) are likely to increase the smoking behavior of employees, as a behavioral coping style [10,11]. Accompanied with underground coal miners, many kinds of physical agents have coexisted in the underground mining environment, such as noise, vibration, temperature and humidity, which are seriously threatening their safety and health. Moreover, various poor occupationally psychosocial factors are prone to increase the levels of occupational stress and psychological distress of underground miners [12,13]. Especially in China, coal resource is the main body of national primary energy production and consumption [14]. There are about six million underground coal miners in China, and most of them often have to face various occupational stressors, such as work overload, low social status, workplace discrimination, strict management, irregular life and work-life interference [13,15]. The above-mentioned risk factors could be considered as sound reasons that are likely to facilitate their cigarette smoking. However, for underground operation, the improvement of external environment would be limited. Therefore, it is needed to look for some factors that could be intervened to reduce smoking behavior from personal or internal perspective.

Psychological capital (PsyCap) is an important psychological resource, and it refers to a positive psychological state that is manifested in the process of individual growth and development. As a higher-order core construct, PsyCap consists of four state-like psychological capacities, including self-efficacy, hope, resilience and optimism [16]. Self-efficacy is a positive belief in one’s work ability to deal with challenging tasks; hope is defined as a positive motivational state directing perseverance towards goals and pathways; resilience refers to one’s positive capacity to bounce back from adversity to attain success; optimism refers to an explanatory style regarding self-attribution for positive events [16]. PsyCap has significantly positive effects on an employee’s work attitudes and behaviors, such as job engagement, satisfaction and performance [17]. It is negatively related to the job stress, burnout, depressive and anxious symptoms of employees and can improve their well-being [18-21]. Yet, to the best of our knowledge, the roles of PsyCap and the components of PsyCap on smoking have not been studied among occupational populations. In the studies of smoking-related factors and interventions, the inhibitory effect of self-efficacy on smoking behavior has been widely confirmed [22,23]. The development and promotion of self-efficacy have become one of the interventions to smoking cessation [24]. Resilience seems to have mixed effects on cigarette smoking. As one of the components of resilience, social competition could contribute to smoking whereas family cohesion is a protective resource to decrease the likelihood of smoking among adolescents [25,26]. For instance, the proportion of current smokers was higher among the pessimists than among the optimists in young Finnish adults [27]. These findings suggest that there could be some associations of PsyCap and the components of PsyCap with smoking behavior among occupational populations, although there are some differences in the specific concepts of positive psychological resources across different fields of research.

In light of the above concerns, the purpose of the present study was to assess the prevalence of cigarette smoking and examine the associations of PsyCap and its components with cigarette smoking among Chinese underground coal miners. In addition, demographic characteristics (age, marital status and education), working factors (job rank and occupational category) and occupational stressors including effort-reward imbalance (ERI) and perceived physical environment (PPE) were considered as possible factors of cigarette smoking in this study.

## Methods

### Study design and sample

A cross-sectional survey was conducted in a coal-mining population from six coal mines in northeast China from July through August of 2013. A stratified multistage cluster sampling design was used in this survey. Within each of these coal mines, eight work teams were randomly selected, including two mining work teams, two tunneling work teams, and one work team from electromechanical maintenance, transportation, ventilation and drainage systems, respectively, according to the institutional settings of underground mining in China. Then, four work groups were selected within mining and tunneling work teams and two work groups were selected within the other four work teams, respectively, using a simple random sampling method without replacement. A work group is the most basic unit of coal mining organization and production in China. In general, there are two or three head miners in each work group, the rest of the group members are staff miners. According to the basic work task, the number of miners in each work group varies from less than 10 to 30 or more. In total, twenty-four work groups were selected in each coal mine. Miners who have engaged in underground work for more than one year from each work group became our study subjects. Twenty-five hundreds of male underground miners were cluster sampled in this study. An additional table file shows this in more detail [see Additional file 1]. Written informed consents were obtained from all participants after they were informed about the study. A set of self-administered questionnaires were completed on paper anonymously in a meeting room at each coal mine when they returned to the surface after a day shift. During the process of completing questionnaires, there was no interference caused by the investigators. In the final analysis, data with missing information concerning any item within questionnaires were excluded. Complete responses were obtained from 1,956 individuals (response rate: 78.2%). The study was approved by the Committee on Human Experimentation of China Medical University, and the study procedures were in accordance with ethical standards.

### Measures

#### Current smoking status

Current smoking status was assessed by the following two questions: (1) have you ever smoked more than 100 cigarettes in your lifetime? (2) had you smoked a cigarette, even a puff in the past 30 days? Respondents were coded as current smokers if they answered “yes” to both questions; otherwise, they were coded to be current nonsmokers that included nonsmokers (answered “no” to the first question) and former smokers (answered “yes” to question and answered “no” to the second question) in this study.

#### PsyCap

The Chinese version of the 24-item Psychological Capital Questionnaire (PCQ) was adopted to measure PsyCap in this study [16]. The PCQ consists of four subscales (self-efficacy, hope, resilience and optimism), and each subscale has six items. For each item, there are six responses with categories ranging from 1 “strongly disagree” to 6 “strongly agree”. Average scores were calculated as the indicators of PsyCap, self-efficacy, hope, resilience and optimism, respectively, and higher scores indicate greater positive psychological states. The Chinese version of PCQ has been widely applied across occupational populations with good reliability and validity in China [19,20,28]. In this study, Cronbach’s alpha of the total scale and self-efficacy, hope, resilience and optimism subscales were 0.94, 0.88, 0.86, 0.86 and 0.80, respectively.

#### Demographic and working factors

Age, marital status, education, job rank and occupational category were obtained in this study. Age was divided into three groups: ≤ 35, 36–45 and ≥ 46 years. Marital status was categorized as single/divorced/widowed/separated and married/cohabiting. Education was categorized as junior high school or under and senior high school/technical secondary school or above. Job rank was categorized as head miner and staff miner. Occupational category was categorized as mining/tunneling and supporting (electromechanical maintenance, transportation, ventilation and drainage).

#### Occupational stressors

ERI was measured using two subscales (effort and reward subscales) from the Chinese version of the Effort-Reward Imbalance scale with good reliability and validity among Chinese occupational groups [28-30]. The effort subscale has six items, and the reward subscale has eleven items. Respondents should initially express their attitude towards some specific work situations (“agree” or “disagree”), and then assess the degree of distress in these situations (from “not distressed” to “very distressed”). The ERI was calculated using the predefined formula: ERI = effort/(reward*0.5454). A higher ERI represents higher level of imbalance. The ERI was divided into tertiles for the analysis with the highest tertile indicating the highest level of occupational stress. In this study, the Cronbach’s alpha for the effort and reward subscales were 0.86 and 0.92, respectively.

PPE was measured using the physical environment subscale derived from the Chinese version of the Occupational Stress Inventory-Revised Edition (OSI-R) [31,32]. This subscale consists of ten items, and each item has five responses with categories ranging from 1 “never” to 5 “very often”. Total score for the physical environment scale was calculated as the indicator of PPE in this study, and higher PPE scores indicate harsher physical environment in workplaces. Also, the PPE score was divided into tertiles for the analysis with the highest tertile indicating the highest level of PPE. Cronbach’s alpha of this scale was 0.79.

### Statistical analysis

The distributions of current smoking among subjects were compared using chi-square test. Differences in continuous variables were examined by t-tests or one-way ANOVAs. Multivariate logistic regression analysis was used to estimate the factors in relation to current smoking. Firstly, Kappa test was used to examine the correlations between categorical independent variables. A kappa value of more than 0.50 indicates agreement among categorical variables, and these variables were adjusted in multivariate analysis. In this study, all Kappa values were < 0.50, and there was no agreement. Then, we examined the associations of PsyCap and its four components with cigarette smoking (model 1 and model 2, respectively). Next, individual variables that included age, marital status, education, job rank, occupational category, ERI and PPE were added in model 3 and model 4. Odds ratios (ORs) and 95% confidence intervals (95% CI) were calculated using current smoker versus nonsmoker. We used −2 log likelihood (−2LL) to compare the goodness-of-fit of each model. All analyses were conducted using SPSS for Windows, Ver. 13.0. Statistical significance was defined as p < 0.05 (two-tailed).

## Results

### Demographic and occupational factors and current smoking status

The demographic characteristics, occupational factors and the proportion of current smokers among subjects are shown in Table 1. The overall smoking prevalence was 52.4%, with the prevalence among the miners who were married/cohabiting being higher (54.1%) than among those who were single/divorced/widowed/separated (44.7%). Smoking prevalence was higher among the miners with lower educational level (59.5%) than those with higher educational level (46.4%). There were positive relationships between both age and PPE in relation to smoking prevalence. However, there was no significant difference in smoking prevalence by job rank, occupational category and ERI level.

**Table 1:** **Demographic and occupational factors and current smoking status**

	**n (%)**	**Current smoker n (%)**	**Current nonsmoker n (%)**	**p value**
All	1956 (100)	1025 (52.4)	931 (47.6)	
Age (years)				< 0.001
≤35	559 (28.6)	241 (43.1)	318 (56.9)	
36–45	752 (38.4)	417 (55.5)	335 (44.5)	
≥46	645 (33.0)	367 (56.9)	278 (43.1)	
Marital status				0.001
Single/divorced/widowed/separated	347 (17.7)	155 (44.7)	192 (55.3)	
Married/cohabiting	1609 (82.3)	870 (54.1)	739 (45.9)	
Education				<0.001
Junior high school or under	897 (45.9)	534 (59.5)	363 (40.5)	
Senior high school/technical secondary school or above	1059 (54.1)	491 (46.4)	568 (53.6)	
Job rank				0.058
Head miner	388 (19.8)	220 (56.7)	168 (43.3)	
Staff miner	1568 (80.2)	805 (51.3)	763 (48.7)	
Occupational category				0.949
Mining/tunneling	1049 (53.6)	549 (52.3)	500 (47.7)	
Supporting	907 (46.4)	476 (52.5)	431 (47.5)	
ERI				0.236
Low	652 (33.3)	324 (49.7)	328 (50.3)	
Intermediate	653 (33.4)	350 (53.6)	303 (46.4)	
High	651 (33.3)	351 (53.9)	300 (46.1)	
PPE				0.009
Low	647 (33.1)	310 (47.9)	337 (52.1)	
Intermediate	657 (33.6)	370 (56.3)	287 (43.7)	
High	652 (33.3)	345 (52.9)	307 (47.1)	

ERI, effort-reward imbalance; PPE, perceived physical environment.

### PsyCap scores and differences in current smoking status, demographic and occupational factors

PsyCap scores and differences in current smoking status, demographic and occupational factors are presented in Table 2. There was no significant difference observed in the levels of PsyCap and its components between current smokers and nonsmokers. The miners aged 36–45 years and head miners reported higher PsyCap and the four components of PsyCap than their counterparts. PsyCap, resilience and optimism among married/cohabiting miners were higher than among those in other marital status. Supporting miners reported higher PsyCap, self-efficacy, resilience and optimism than mining/tunneling miners did. Higher levels of ERI were associated with lower PsyCap and its four components. There was no significant difference determined in PsyCap and the four components of PsyCap by education and PPE levels.

**Table 2:** **PsyCap scores and differences in current smoking status, demographic and occupational factors**

	**PsyCap Mean (SD)**	**Self-efficacy Mean (SD)**	**Hope Mean (SD)**	**Resilience Mean (SD)**	**Optimism Mean (SD)**
Smoking status					
Current smoker	4.29 (0.77)	4.28 (0.90)	4.27 (0.86)	4.31 (0.85)	4.29 (0.93)
Current nonsmoker	4.27 (0.81)	4.26 (0.93)	4.27 (0.90)	4.26 (0.93)	4.32 (0.94)
Age (years)					
≤35	4.17 (0.80)**	4.17 (0.96)**	4.18 (0.90)**	4.18 (0.90)**	4.15 (0.98)**
36–45	4.36 (0.76)	4.36 (0.85)	4.33 (0.86)	4.36 (0.88)	4.40 (0.89)
≥46	4.29 (0.79)	4.25 (0.94)	4.28 (0.88)	4.30 (0.88)	4.34 (0.93)
Marital status					
Single/Divorced/Widowed/Separated	4.20 (0.80)*	4.20 (0.95)	4.22 (0.90)	4.19 (0.88)*	4.20 (0.95)*
Married/Cohabiting	4.29 (0.78)	4.29 (0.90)	4.28 (0.88)	4.31 (0.89)	4.33 (0.93)
Education					
Junior high school or under	4.26 (0.74)	4.24 (0.87)	4.25 (0.83)	4.27 (0.84)	4.29 (0.90)
Senior high school/Technical secondary school or above	4.30 (0.82)	4.30 (0.95)	4.29 (0.92)	4.30 (0.93)	4.32 (0.96)
Job rank					
Head miner	4.40 (0.80)	4.37 (0.91)	4.39 (0.86)	4.41 (0.89)	4.45 (0.91)
Staff miner	4.25 (0.78)**	4.25 (0.91)*	4.24 (0.88)**	4.26 (0.89)**	4.27 (0.94)**
Occupational category					
Mining/Tunneling	4.24 (0.81)**	4.21 (0.94)**	4.24 (0.90)	4.24 (0.90)*	4.26 (0.96)*
Supporting	4.34 (0.76)	4.34 (0.88)	4.31 (0.84)	4.34 (0.87)	4.36 (0.90)
ERI					
Low	4.47 (0.76)	4.43 (0.86)	4.47 (0.84)	4.45 (0.85)	4.52 (0.88)
Intermediate	4.31 (0.76)	4.30 (0.91)	4.29 (0.85)	4.31 (0.85)	4.36 (0.87)
High	4.07 (0.80)**	4.08 (0.94)**	4.04 (0.89)**	4.11(0.93)**	4.04 (0.99)**
PPE					
Low	4.31 (0.76)	4.27 (0.88)	4.32 (0.86)	4.31 (0.86)	4.36 (0.89)
Intermediate	4.28 (0.75)	4.26 (0.85)	4.27 (0.84)	4.28 (0.85)	4.31 (0.90)
High	4.26 (0.85)	4.29 (1.00)	4.23 (0.94)	4.28 (0.95)	4.25 (1.00)

PsyCap, psychological capital; SD, standard deviation; ERI, effort-reward imbalance; PPE, perceived physical environment.

*p < 0.05, **p < 0.01.

### Multivariate logistic regression results predicting current smoking

The results derived from the multivariate logistic regression analyses of factors associated with current cigarette smoking are shown in Table 3. Without controlling for demographic and occupational variables, model 1 indicated that PsyCap had no association with smoking prevalence; while resilience had a positive association with current smoking, and optimism decreased the prevalence of current smoking (model 2). After controlling for demographic and occupational variables, PsyCap was still not associated with smoking prevalence in model 3. Smoking prevalence was higher among the miners with lower education level than among those with higher education level (OR: 1.53, 95% CI: 1.23–1.89). Compared with the miners in the first tertile (low) of PPE, the ORs of smoking for the miners in the second tertile (intermediate) and the third tertile (high) of PPE were 1.39 (95% CI: 1.11–1.75) and 1.22 (95% CI: 0.97–1.54), respectively. When PsyCap was replaced with its four components in model 4, compared with the miners in the first tertile (low) of resilience, the ORs of smoking for the miners in the second tertile (intermediate) and the third tertile (high) of resilience were 1.30 (95% CI: 0.99–1.70) and 1.58 (95% CI: 1.13–2.20), respectively. Compared with the miners in the first tertile (low) of optimism, the ORs of smoking for the miners in the second tertile (intermediate) and third tertile (high) of optimism were 0.79 (95% CI: 0.61–1.03) and 0.69 (95% CI: 0.51–0.92), respectively. The associations of demographic and occupational variables with smoking prevalence showed no significant changes in model 4.

**Table 3:** **Multivariate logistic regression results predicting current smoking**

	**Model 1 OR (95% CI)**	**Model 2 OR (95% CI)**	**Model 3 OR (95% CI)**	**Model 4 OR (95% CI)**
Age (years)				
≤35			1	1
36–45			1.27 (0.97-1.66)	1.29 (0.99-1.69)
≥46			1.26 (0.95-1.69)	1.29 (0.97-1.73)
Marital status				
Single/Divorced/Widowed/Separated vs. Married/Cohabiting			0.92 (0.71-1.20)	0.95 (0.73-1.24)
Education				
Junior high school or under vs. Senior high school/Technical secondary school or above			1.53 (1.23-1.89)	1.54 (1.24-1.91)
Job rank				
Staff miner vs. Head miner			0.83 (0.66-1.04)	0.83 (0.66-1.04)
Occupational category				
Mining/Tunneling vs. Supporting			0.93 (0.77-1.12)	0.93 (0.77-1.12)
ERI				
Low			1	1
Intermediate			1.13 (0.90-1.41)	1.12 (0.90-1.40)
High			1.11 (0.88-1.40)	1.10 (0.87-1.40)
PPE				
Low			1	1
Intermediate			1.39 (1.11-1.75)	1.39 (1.11-1.74)
High			1.22 (0.97-1.54)	1.21 (0.95-1.53)
PsyCap				
Low	1		1	
Intermediate	1.13 (0.91-1.40)		1.12 (0.90-1.40)	
High	1.01 (0.81-1.25)		1.00 (0.79-1.25)	
Self-efficacy				
Low		1		1
Intermediate		1.13 (0.88-1.45)		1.12 (0.87-1.45)
High		0.97 (0.71-1.31)		0.99 (0.72-1.35)
Hope				
Low		1		1
Intermediate		1.20 (0.92-1.55)		1.23 (0.94-1.61)
High		0.91 (0.65-1.27)		0.90 (0.64-1.27)
Resilience				
Low		1		1
Intermediate		1.31 (1.01-1.71)		1.30 (0.99-1.70)
High		1.61 (1.16-2.23)		1.58 (1.13-2.20)
Optimism				
Low		1		1
Intermediate		0.81 (0.63-1.05)		0.79 (0.61-1.03)
High		0.70 (0.52-0.93)		0.69 (0.51-0.92)
−2 Log likelihood	2705.56	2687.44	2650.21	2631.63

PsyCap, psychological capital; OR, odds ratio; CI, confidence intervals; ERI, effort-reward imbalance; PPE, perceived physical environment.

## Discussion

In view of production safety, there are strict tobacco control measures in underground coal mining. Perhaps, to a certain extent, these measures could reduce the smoking behavior of employees in workplaces. However, the present study found that the overall smoking prevalence was 52.4% among underground coal miners in northeast China. It was consistent with the previous studies on underground coal miners from Xuzhou Coal Mining Group in China (56.3%) [6], both surface and underground coal miners in northern China (54.0%) [33], male employees (54.2%) [9] and leaders of organization (54.1%) in different workplaces [3]. This prevalence was lower than the national survey of agricultural workers and machine operators in China [3] and underground coal miners who residing in Zonguldak city of Turkey (66.3%) [5], but higher than health care personnel and teaching staff in Chinese males [3]. In addition, the smoking prevalence was also higher than underground coal miners from the US (22.0%) [34] and the southern part of India (less than 20.0%) [35]. Due to the special working and living conditions of underground coal miners, smoking is one of the highly prevalent health risk behaviors. Therefore, in-depth research on the related factors of cigarette smoking should be conducted to develop effective coping strategies and measures in this occupational population.

Although no comparable study has been conducted, several previous studies in other countries indicated that as positive psychological resources, self-efficacy and optimism were significantly and negatively associated with smoking behavior, respectively, while resilience had mixed effects on cigarette smoking among different populations as mentioned above. In this study, we found that PsyCap had mixed effects on smoking behavior. Resilience was positively associated with the smoking behavior of underground coal miners, whereas optimism, as a protective role, could reduce smoking behavior. At work, resilience could help employees overcome various difficulties and quickly extricate themselves from adversity to defeat for the impetus to improve performance [16,17]. Resilient people are more able to cope with high occupational stress, and less likely to suffer from psychological distress [18,19,28]. However, although an increasing number of studies have confirmed the positive effects of resilience on individual and organizational outcomes [36], this study found that resilience could contribute to the cigarette smoking among underground coal miners. One reason for this is probably because that in response to competitive pressures and challenges at work, employees with high resilience are more likely to smoke cigarettes due to the physiological and psychological benefits of tobacco use that is often used as a way of coping with various stresses. According to the concept of PsyCap, resilience is designed to measure employees’ psychological and behavioral responses to stress and adversity at work. The results of previous studies indicated that as one of the components of resilience, social competition could contribute to adolescent smoking [25,26]. Moreover, according to Chinese culture, smoking is perceived as an important component of social activity and a way of interpersonal communication [37]. Improving communication with others is a way to improve the resilience which might partly explain the relationship between resilience and smoking [38]. Accordingly, this finding prompted us to concern more about the various behavioral changes of employees, especially stress-related health risk behaviors, when some effective measures are implemented to develop their resilience in order to obtain its positive effects. If necessary, employees should be given powerful organizational or social supports to reduce the prevalence of health risk behaviors. Fortunately, consistent with many previous studies [27], optimism is a protective factor of smoking behavior in this study. Optimistic employees are less distressed by occupational stressors and often perceive less stress, and most of them will facilitate positive behavior lifestyles conducive to their health. Investment in optimism development will thus have a dual role in organizational behavior outcomes and personal health-related behaviors. For example, in a sample of workers in manufacturing sector, the level of optimism was raised through development of an optimism subculture and implementation of a goal setting process [39]. However, self-efficacy and hope had no significant impacts on cigarette smoking in the current study. One possible reason could be that the associations of self-efficacy and hope with mental health problems (e.g., depressive symptoms) were weak or not significant compared with that of resilience and optimism among occupational populations [28]. This suggests that the positive effects of self-efficacy and hope on relieving mental health problems could be limited in workplaces, although these mental health issues are important factors contributing to smoking. In addition, due to the opposite effects of resilience and optimism, the integrated effect of PsyCap on cigarette smoking was not significant.

In the univariate analysis of this study, married/cohabiting miners reported significantly higher current smoking compared with those in other marital status. Taking positive psychological variables into account, it was found that married/cohabiting miners showed significantly higher levels of resilience and optimism. As a result, no significant relationship between marital status and current smoking in multivariate analysis is probably due to the opposite effects of resilience and optimism on smoking. This result suggests that significant changes in the levels of resilience and optimism might be a potential mechanism for clarifying the link between marital status and smoking. Similarly, the relationship between age and smoking might also be influenced by significant differences in resilience and optimism levels. In addition, education had a significant impact on smoking behavior. Low education level was a vital risk factor of smoking among underground coal miners, which was consistent with other studies [40,41]. The miners with lower education level had higher prevalence of cigarette smoking than those with higher education level in this study. People with higher education level might have higher awareness of the serious harms of tobacco use and stronger responsibility for their own health and safety compared with those with lower level of education, which would promote the development and persistence of health and safety-related behaviors.

ERI had no effect on cigarette smoking in the current study. The results of several previous studies on the associations between ERI and smoking were inconsistent. A cross-sectional study of 179 middle-aged male middle-managers in a large car-producing enterprise showed that ERI was associated with cigarette smoking [42], whereas the other two studies found that ERI was not related to smoking in men, but there were significant associations in women [10,43]. Potential reason for this inconsistency might be because occupation and sample size differences in many earlier studies possibly decreased the likelihood of detecting significant associations. For many underground coal miners, the main motivation for mining is relatively higher income. As an important part of work reward, high income level tends to reduce the ERI level of our sample. In addition, ERI had significant negative impacts on the levels of PsyCap and the components of PsyCap in this study. Therefore, the potential confounding effect of ERI should been taken into account when we try to explore the effects of PsyCap and its four components on cigarette smoking. PPE was found to be slightly associated with smoking behavior. As an important source of occupational stress among underground coal miners, the high perception of physical environment could result in psychological distress [32], which is prone to increase their smoking. Effective measures should be taken to reduce the level of PPE in this occupational population, though the improvement of external environment would probably be limited.

This is the first study to confirm the effects of PsyCap and the components of PsyCap on cigarette smoking. The findings from our study might have theoretical implication for developing some potentially effective interventions on the prevention and reduction of cigarette smoking among Chinese underground coal miners. Effective strategies and measures should be implemented among underground coal miners to improve education level and relieve PPE. Additionally, resilience should be carefully promoted despite its positive effects on individual and organizational outcomes in workplaces, given its potential contribution to smoking behavior. In view of the positive effects of optimism on both organizational and personal behaviors, managers should encourage underground coal miners to regard past failures as valuable experience, enhance their ability to find out various opportunities for success and develop a positive attribution style [44,45].

However, there are several limitations in this study. First, the cross-sectional design of the present study is impossible to draw causal relationships among study variables. Longitudinal studies are required to confirm these findings. Second, only the underground coal miners from a coal-mining population in northeast China were sampled in this study. However, the large sample size and high effective response rate of this study could contribute to the generalization of our findings. Third, the unique use of self-report measures might influence the associations among study variables. Some effective process control measures were carried out to reduce the common-method bias, such as protecting the anonymity of respondents and assuring respondents that there were no right or wrong answers for their opinions. Finally, more occupationally psychological and social factors (e.g., work-life interference, organizational support, social capital and job satisfaction) were not addressed in this study, which should be considered in further studies.

## Conclusions

More than half of the underground coal miners were current smokers, which indicated that cigarette smoking might be a common health risk behavior in this occupational population. High resilience and PPE, together with low education were the risk factors of smoking behavior, whereas high optimism was a protective factor. The integrated effect of PsyCap on smoking was not significant due to the opposite effects of resilience and optimism. Consequently, the behavioral changes of employees should be given more attention when some effective measures are implemented to develop their resilience in order to obtain its positive effects.

## Additional file

Additional file 1:
**Population stratification and sample size in each selected coal mine, work team and work group.** Description of table: The table shows population stratification and the type and the number of participants and complete responses of each sampled work group in this study.

## Abbreviations

−2LL: −2 log likelihood
CI: Confidence intervals
ERI: Effort-reward imbalance
ORs: Odds ratios
OSI-R: Occupational stress inventory-revised edition
PPE: Perceived physical environment
PsyCap: Psychological capital
PCQ: Psychological capital questionnaire
SD: Standard deviation

## References

[1] WHO (2013). WHO report on The global tobacco epidemic, 2013. Enforcing bans on tobacco advertising, promotion and sponsorship.

[2] The Ministry of Health (MOH) of the People’s Republic of China (2012). China report on the health hazards of smoking.

[3] Li Q, Hsia J, Yang G (2011). Prevalence of smoking in China in 2010. N Engl J Med.

[4] Centers for Disease Control and Prevention (CDC) (2011). Current cigarette smoking prevalence among working adults-United States, 2004–2010. MMWR Morb Mortal Wkly Rep.

[5] Unalacak M, Altin R, Kart L, Tor M, Ornek T, Altunel H (2004). Smoking prevalence, behaviour and nicotine addiction among coal workers in Zonguldak, Turkey. J Occup Health.

[6] Song Z, Zhang Y, Zhang M (2013). Analysis on occupational health status of exposure workers in 2 coal mines of Xuzhou Coal Mining Group. Occup and Health.

[7] Veiga LH, Amaral EC, Colin D, Koifman S (2006). A retrospective mortality study of workers exposed to radon in a Brazilian underground coal mine. Radiat Environ Biophys.

[8] Legleye S, Baumann M, Peretti-Watel P, Beck F, Chau N (2011). Gender and age disparities in the associations of occupational factors with alcohol abuse and smoking in the French working population. Rev Epidemiol Sante Publique.

[9] Gao J, Nehl EJ, Fu H, Jia Y, Liu X, Zheng P (2013). Workplace social capital and smoking among Chinese male employees: a multi-level, cross-sectional study. Prev Med.

[10] Kouvonen A, Kivimäki M, Virtanen M, Pentti J, Vahtera J (2005). Work stress, smoking status, and smoking intensity: an observational study of 46,190 employees. J Epidemiol Community Health.

[11] Lawrence D, Mitrou F, Zubrick SR (2011). Non-specific psychological distress, smoking status and smoking cessation: United States National Health Interview Survey 2005. BMC Public Health.

[12] McLean KN (2012). Mental health and well-being in resident mine workers: out of the fly-in fly-out box. Aust J Rural Health.

[13] Yao LF, Yao SF (2007). Coping style and psychological health in coal mine workers. Zhonghua Lao Dong Wei Sheng Zhi Ye Bing Za Zhi.

[14] Liu FD, Pan ZQ, Liu SL, Chen L, Ma JZ, Yang ML, Wang NP (2007). The estimation of the number of underground coal miners and the annual dose to coal miners in China. Health Phys.

[15] Yang HX, Wang XY, Song JX, Wang QL (2011). Investigation on relationships among social support, life events and psychological health of coal mine workers. Chin Nurs Res.

[16] Luthans F, Avolio BJ, Avey JB, Norman SM (2007). Positive psychological capital: measurement and relationship with performance and satisfaction. Pers Psychol.

[17] Avey JB, Reichard RJ, Luthans F, Mhatre KH (2011). Meta-analysis of the impact of positive psychological capital on employee attitudes, behaviors, and performance. Hum Resour Dev Q.

[18] Avey JB, Luthans F, Jensen SM (2009). Psychological capital: a positive resource for combating employee stress and turnover. Hum Resour Manage.

[19] Wang Y, Liu L, Wang J, Wang L (2012). Work-family conflict and burnout among Chinese doctors: the mediating role of psychological capital. J Occup Health.

[20] Liu L, Pang R, Sun W, Wu M, Qu P, Lu C, Wang L (2013). Functional social support, psychological capital, and depressive and anxiety symptoms among people living with HIV/AIDS employed full-time. BMC Psychiatry.

[21] Avey JB, Luthans F, Smith RM, Palmer NF (2010). Impact of positive psychological capital on employee well-being over time. J Occup Health Psychol.

[22] Cupertino AP, Berg C, Gajewski B, Hui SK, Richter K, Catley D, Ellerbeck EF (2012). Change in self-efficacy, autonomous and controlled motivation predicting smoking. J Health Psychol.

[23] Gwaltney CJ, Metrik J, Kahler CW, Shiffman S (2009). Self-efficacy and smoking cessation: a meta-analysis. Psychol Addict Behav.

[24] Hyde J, Hankins M, Deale A, Marteau TM (2008). Interventions to increase self-efficacy in the context of addiction behaviours: a systematic literature review. J Health Psychol.

[25] Veselska Z, Geckova AM, Orosova O, Gajdosova B, van Dijk JP, Reijneveld SA (2009). Self-esteem and resilience: the connection with risky behavior among adolescents. Addict Behav.

[26] Skrove M, Romundstad P, Indredavik MS (2013). Resilience, lifestyle and symptoms of anxiety and depression in adolescence: the Young-HUNT study. Soc Psychiatry Psychiatr Epidemiol.

[27] Kelloniemi H, Ek E, Laitinen J (2005). Optimism, dietary habits, body mass index and smoking among young Finnish adults. Appetite.

[28] Liu L, Chang Y, Fu J, Wang J, Wang L (2012). The mediating role of psychological capital on the association between occupational stress and depressive symptoms among Chinese physicians: a cross-sectional study. BMC Public Health.

[29] Siegrist J (1996). Adverse health effects of high-effort/low-reward conditions. J Occup Health Psychol.

[30] Yang WJ, Li J (2004). Measurement of psychosocial factors in work environment: application of two models of occupational stress. Zhong hua Lao Dong Wei Sheng Zhi Ye Bing Za Zhi.

[31] Osipow SH (1998). Occupational stress inventory revised edition (professional manual).

[32] Liu L, Wang L, Chen J (2014). Prevalence and associated factors of depressive symptoms among Chinese underground coal miners. Biomed Res Int.

[33] Xu G, Pang D, Liu F, Pei D, Wang S, Li L (2012). Prevalence of low back pain and associated occupational factors among Chinese coal miners. BMC Public Health.

[34] Wang ML, Beeckman-Wagner LA, Wolfe AL, Syamlal G, Petsonk EL (2013). Lung-function impairment among US underground coal miners, 2005 to 2009: geographic patterns and association with coal workers’ pneumoconiosis. J Occup Environ Med.

[35] Kunar BM, Bhattacherjee A, Chau N (2010). A matched case–control study of occupational injury in underground coalmine workers. J S Afr Inst Min Metall.

[36] Spangler NW, Koesten J, Fox MH, Radel J (2012). Employer perceptions of stress and resilience intervention. J Occup Environ Med.

[37] Rich ZC, Xiao S (2012). Tobacco as a social currency: cigarette gifting and sharing in China. Nicotine Tob Res.

[38] Zuo X, Peng L, Li M, Yao Z, Li J (2011). Impact of resilience training on positive /negative emotions and heart rate variability in surface ship soldiers with different resilience. J Third Mil Med Univ.

[39] Green KW, Medlin B, Whitten D (2004). Developing optimism to improve performance: an approach for the manufacturing sector. Ind Manage Data Syst.

[40] Chin DL, Hong O, Gillen M, Bates MN, Okechukwu CA (2012). Cigarette smoking in building trades workers: the impact of work environment. Am J Ind Med.

[41] Jain NB, Hart JE, Smith TJ, Garshick E, Laden F (2006). Smoking behavior in trucking industry workers. Am J Ind Med.

[42] Peter R (1995). Job stressors, coping characteristics, and the development of coronary heart disease (CHD): Results from two studies. Psychologische Beitrage.

[43] Kouvonen A, Kivimäki M, Virtanen M, Heponiemi T, Elovainio M, Pentti J, Linna A, Vahtera J (2006). Effort-reward imbalance at work and the co-occurrence of lifestyle risk factors: cross-sectional survey in a sample of 36,127 public sector employees. BMC Public Health.

[44] Luthans F, Avey JB, Patera JL (2008). Experimental analysis of a web-based training intervention to develop positive psychological capital. Acad Manag Learn Edu.

[45] Wen L, Qi S (2009). Experimental research on the effect of intervention to psychological capital of employee. Chin J Health Psychol.

