# Supplementary material for: Associations of psychological capital, demographic and occupational factors with cigarette smoking among Chinese underground coal miners
Source: BMC Public Health. 2015 Jan 21;15:20. doi: 10.1186/s12889-015-1349-6 (PMC4311445; doi:10.1186/s12889-015-1349-6)
Supplement: Additional file 1: — Population stratification and sample size in each selected coal mine, work team and work group. Description of table: The table shows population stratification and the type and the number of participants and complete responses of each sampled work group in this study. [file 12889_2015_1349_MOESM1_ESM.doc]

**Additional file 1: Table S1: Population stratification and sample size in each selected coal mine, work team and work group**

| Coal mines | Work teams | Work groups | | | | Total |
| --- | --- | --- | --- | --- | --- | --- |
|  |  | 1 | 2 | 3 | 4 |  |
|  |  | N (n) | N (n) | N (n) | N (n) | N (n) |
| Da Long |  |  |  |  |  | 406 (320) |
|  | Mining/Tunneling |  |  |  |  | 242 (183) |
|  | Mining 1 | 16 (12) | 19 (14) | 13 (9) | 13 (10) |  |
|  | Mining 2 | 15 (11) | 17 (12) | 15 (11) | 19 (16) |  |
|  | Tunneling 1 | 17 (13) | 22 (18) | 13 (10) | 12 (8) |  |
|  | Tunneling 2 | 12 (10) | 14 (11) | 11 (8) | 14 (10) |  |
|  | Supporting |  |  |  |  | 164 (137) |
|  | Electromechanical maintenance | 21 (16) | 23 (19) |  |  |  |
|  | Transportation | 25 (22) | 22 (19) |  |  |  |
|  | Ventilation | 18 (15) | 16 (12) |  |  |  |
|  | Drainage | 21 (19) | 18 (15) |  |  |  |
| Da Ming |  |  |  |  |  | 364 (286) |
|  | Mining/Tunneling |  |  |  |  | 213 (164) |
|  | Mining 1 | 14 (10) | 14 (11) | 12 (9) | 13 (11) |  |
|  | Mining 2 | 11 (9) | 12 (8) | 15 (11) | 16 (13) |  |
|  | Tunneling 1 | 17 (13) | 16 (13) | 13 (10) | 12 (8) |  |
|  | Tunneling 2 | 12 (10) | 13 (10) | 11 (8) | 12 (10) |  |
|  | Supporting |  |  |  |  | 151 (122) |
|  | Electromechanical maintenance | 17 (14) | 23 (19) |  |  |  |
|  | Transportation | 20 (17) | 19 (15) |  |  |  |
|  | Ventilation | 16 (12) | 14 (11) |  |  |  |
|  | Drainage | 22 (18) | 20 (16) |  |  |  |
| Da Xing |  |  |  |  |  | 418 (332) |
|  | Mining/Tunneling |  |  |  |  | 225 (170) |
|  | Mining 1 | 15 (11) | 16 (13) | 12 (9) | 12 (10) |  |
|  | Mining 2 | 13 (11) | 17 (14) | 12 (8) | 13 (9) |  |
|  | Tunneling 1 | 13 (10) | 17 (12) | 18 (14) | 12 (8) |  |
|  | Tunneling 2 | 11 (8) | 13 (10) | 16 (12) | 15 (11) |  |
|  | Supporting |  |  |  |  | 193 (162) |
|  | Electromechanical maintenance | 24 (20) | 26 (21) |  |  |  |
|  | Transportation | 28 (25) | 26 (22) |  |  |  |
|  | Ventilation | 22 (18) | 19 (15) |  |  |  |
|  | Drainage | 23 (20) | 25 (21) |  |  |  |
| Xiao Ming |  |  |  |  |  | 397 (312) |
|  | Mining/Tunneling |  |  |  |  | 216 (161) |
|  | Mining 1 | 15 (11) | 13 (10) | 10 (7) | 14 (11) |  |
|  | Mining 2 | 16 (12) | 13 (9) | 12 (9) | 15 (10) |  |
|  | Tunneling 1 | 11 (8) | 14 (11) | 16 (12) | 11 (8) |  |
|  | Tunneling 2 | 12 (9) | 15 (12) | 13 (10) | 16 (12) |  |
|  | Supporting |  |  |  |  | 181 (151) |
|  | Electromechanical maintenance | 22 (19) | 20 (16) |  |  |  |
|  | Transportation | 26 (21) | 23 (18) |  |  |  |
|  | Ventilation | 23 (19) | 19 (16) |  |  |  |
|  | Drainage | 25 (21) | 23 (21) |  |  |  |
| Xiao Nan |  |  |  |  |  | 432 (340) |
|  | Mining/Tunneling |  |  |  |  | 234 (177) |
|  | Mining 1 | 16 (12) | 23 (18) | 13 (10) | 16 (11) |  |
|  | Mining 2 | 11 (8) | 14 (10) | 12 (9) | 13 (10) |  |
|  | Tunneling 1 | 16 (12) | 15 (11) | 12 (11) | 16 (13) |  |
|  | Tunneling 2 | 13 (9) | 16 (12) | 13 (9) | 15 (12) |  |
|  | Supporting |  |  |  |  | 198 (163) |
|  | Electromechanical maintenance | 26 (22) | 23 (19) |  |  |  |
|  | Transportation | 23 (19) | 27 (22) |  |  |  |
|  | Ventilation | 26 (21) | 23 (19) |  |  |  |
|  | Drainage | 26 (21) | 24 (20) |  |  |  |
| Xiao Qing |  |  |  |  |  | 483 (366) |
|  | Mining/Tunneling |  |  |  |  | 270 (194) |
|  | Mining 1 | 20 (16) | 25 (21) | 15 (10) | 17 (12) |  |
|  | Mining 2 | 14 (10) | 15 (10) | 13 (8) | 16 (12) |  |
|  | Tunneling 1 | 15 (11) | 17 (12) | 14 (9) | 22 (16) |  |
|  | Tunneling 2 | 17 (12) | 14 (10) | 16 (11) | 20 (14) |  |
|  | Supporting |  |  |  |  | 213 (172) |
|  | Electromechanical maintenance | 28 (23) | 25 (20) |  |  |  |
|  | Transportation | 30 (25) | 27 (21) |  |  |  |
|  | Ventilation | 26 (21) | 24 (20) |  |  |  |
|  | Drainage | 27 (22) | 26 (20) |  |  |  |
| Total |  |  |  |  |  | 2500 (1956) |
|  | Mining/Tunneling |  |  |  |  | 1400 (1049) |
|  | Supporting |  |  |  |  | 1100 (907) |
